# Seroprevalence of Swine Influenza A Virus (swIAV) Infections in Commercial Farrow-to-Finish Pig Farms in Greece

**DOI:** 10.3390/vetsci10100599

**Published:** 2023-09-30

**Authors:** Vasileios G. Papatsiros, Georgios I. Papakonstantinou, Eleftherios Meletis, Konstantinos Koutoulis, Zoi Athanasakopoulou, Georgios Maragkakis, Georgia Labronikou, Ilias Terzidis, Polychronis Kostoulas, Charalambos Billinis

**Affiliations:** 1 Clinic of Medicine, Faculty of Veterinary Medicine, University of Thessaly, 43100 Karditsa, Greece; geopapak@vet.uth.gr (G.I.P.); gmaragkakis@vet.uth.gr (G.M.); 2 Faculty of Public and One Health, University of Thessaly, 43100 Karditsa, Greece; elmeletis@uth.gr (E.M.); pkost@uth.gr (P.K.); 3 Department of Poultry Diseases, Faculty of Veterinary Science, School of Health Sciences, University of Thessaly, 43100 Karditsa, Greece; kkoutoulis@uth.gr; 4 Department of Microbiology and Parasitology, Faculty of Veterinary Medicine, University of Thessaly, 43100 Karditsa, Greece; zathanas@uth.gr (Z.A.); billinis@uth.gr (C.B.); 5 Swine Technical Support, Hipra Hellas SA, 10441 Athens, Greece; georgia.labronikou@hipra.com (G.L.); ilias.terzidis@hipra.com (I.T.)

**Keywords:** pigs, swine influenza virus, antibodies, seroprevalence, Greece

## Abstract

**Simple Summary:**

Our study aimed to assess the seroprevalence of Swine Influenza Viruses (swIAVs) in commercial pig farms in Greece. A total of 1416 blood samples were collected from breeding animals (gilts and sows) and pigs aged 3 weeks to market age from 40 different swIAV vaccinated and unvaccinated commercial farrow-to-finish pig farms. Of the total 1416 animals sampled, 498 were seropositive, indicating that the virus circulates in both vaccinated (54% seroprevalence) and unvaccinated Greek pig farms (23% seroprevalence). In addition, maternally derived antibody (MDA) levels in pigs at 4 and 7 weeks of age were lower in unvaccinated farms than in vaccinated farms. In conclusion, our results underscore the importance of vaccination for the prevention of swIAV infections in commercial farrow-to-finish pig farms.

**Abstract:**

Swine influenza is a highly contagious respiratory disease caused by influenza A virus infection. Pigs play an important role in the overall epidemiology of influenza because of their ability to transmit influenza viruses of avian and human origin, which plays a potential role in the emergence of zoonotic strains with pandemic potential. The aim of our study was to assess the seroprevalence of Swine Influenza Viruses (swIAVs) in commercial pig farms in Greece. A total of 1416 blood samples were collected from breeding animals (gilts and sows) and pigs aged 3 weeks to market age from 40 different swIAV vaccinated and unvaccinated commercial farrow-to-finish pig farms. For the detection of anti-SIV antibodies, sera were analyzed using an indirect ELISA kit CIVTEST SUIS INFLUENZA^®^, Hipra (Amer, Spain). Of the total 1416 animals tested, 498 were seropositive, indicating that the virus circulates in both vaccinated (54% seroprevalence) and unvaccinated Greek pig farms (23% seroprevalence). In addition, maternally derived antibody (MDA) levels were lower in pigs at 4 and 7 weeks of age in unvaccinated farms than in vaccinated farms. In conclusion, our results underscore the importance of vaccination as an effective tool for the prevention of swIAV infections in commercial farrow-to-finish pig farms.

## 1. Introduction

Swine influenza is a viral infectious disease that has a major impact on the swine industry worldwide, mainly because it is one of the most common respiratory diseases in commercial pig herds [1]. The etiological agent of swine influenza is swine influenza A virus (swIAV), which belongs to the Orthomyxoviridae family. It has three different genotypes (H1N1, H2N1, and H3N2) [2,3], which contain genetic components derived from both avian and human influenza viruses, resulting in different lineages depending on geographic location [4,5,6]. SwIAV is endemic in several regions of high pig density, while epidemic outbreaks often occur in naïve pig herds [7]. In addition, swIAV is involved in porcine respiratory disease complex (PRCD), which is associated with important economic losses [7,8,9]. SwIAV is considered an important pathogen for animal and public health [1,10,11]. Pigs are susceptible to both human and avian influenza viruses [12] and may play the role of a “mixing vessel” for the emergence of a new influenza virus through genetic reassortment [13].

SwIAV causes respiratory infections in pigs worldwide [14,15,16], which are associated with high morbidity [3] and significant economic consequences in commercial pig farms [17,18] due to decreased growth performance and increased costs for the prevention and treatment of secondary bacterial or viral pulmonary infections [3,19,20,21]. Clinical signs of SwIAV infection in pigs include pyrexia (40.5–42 °C), anorexia, lethargy, respiratory signs (sneezing, conjunctivitis, cough, nasal discharge, labored breathing), broncho-interstitial pneumonia, abortions, and weight loss [1,3,22,23]. High morbidity and low mortality rates are observed in naïve herds [24].

Previously, it was assumed that the new introduction of swIAV into a pig herd might be associated with clinical signs and high morbidity rates [1]. However, swIAV appears to be more widespread in pigs than previously thought [7]. To understand the epidemiological processes responsible for the endemic persistence of swIAV in pig herds, a quantitative approach to the parameters of virus transmission in a swine population is needed. Field studies suggest an interaction between the immune status of pigs at the time of infection and the spread of swIAV between batches, resulting in persistent infections on farms [25,26]. The majority of field cases are subclinical, while the prevalence of clinical cases is low [2,27,28]. However, persistent swIAV infections have been reported after an acute outbreak [29], while endemic infection in pig farms has never been reported [1,7]. Apart from the picture of a classic epidemic outbreak, there is very little literature evidence of swIAV seroprevalence in commercial pig farms.

Knowledge of the epidemiology of SwIAV is needed for the design of cost-effective control strategies based on vaccination to limit the spread of SwIAV in commercial pig farms. SwIAV occurs in the main pig-rearing regions of Greece, but few data are available on the swIAV seroprevalence in commercial Greek pig herds [30]. Against this background, we aimed to determine the seroprevalence of swIAV in commercial pig farms in Greece.

## 2. Materials and Methods

### 2.1. Sampled Pig Farms

**Figure 1:**
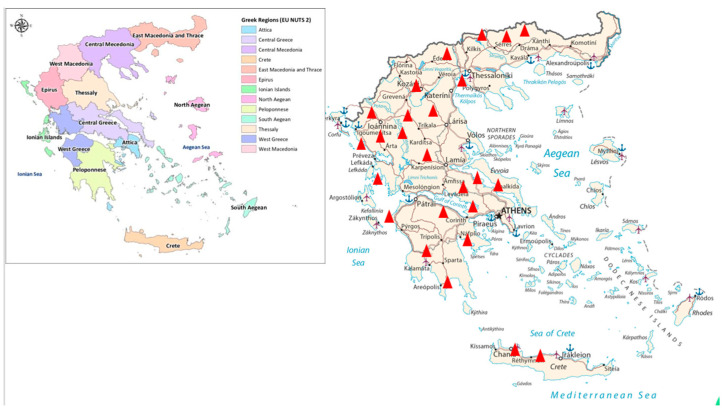
Map of the study region indicating the sampling sites (red triangles) in different regions of Greece, especially in areas with a high density of pigs.

This study was conducted between March 2019 and April 2023, and the included commercial farrow-to-finish pig farms were located in different regions of Greece, especially in areas with a high density of pigs such as Northern Greece [(Drama, Serres, Thessaloniki, Chalkidiki, Pieria (Katerini)], Thessaly (Trikala, Larissa, Volos), Western Greece [Epirus (Ioannina, Filippiada, Arta), Aitoloakarnania (Agrinio)], Southern Greece [Peloponnese (Korinthos, Ileia, Epidauros, Neapoli, Sparta)], Central Greece (Lamia, Viotia, Chalkida, Attica), and Crete (Rethymno, Irakleio) (Figure 1).

Selection criteria for this study were (a) the geographic location of each commercial farm, including farms from the regions with the largest pig production in Greece; (b) the application or absence of swIAV vaccination; and (c) voluntary owner consent to participate in the study. None of the commercial pig farms reported clinical signs of respiratory disease resembling swIAV infection. All study pig farms that were vaccinated against swIAV performed a similar vaccination scheme. Specifically, sows were vaccinated with inactivated vaccines against swine influenza caused by subtypes H1N1, H3N2, and H1N2 (Respiporc^®^ FLU3, Ceva Animal Health, France) or H1N1 and H3N2 (Gripork^®^, Hipra, Spain). Vaccination protocols typically included the initial immunization of gilts, followed by the vaccination of sows. Gilts were initially vaccinated twice (e.g., on the 140th and 170th day of age) before their first artificial insemination. A mass vaccination was applied in all sows every 4 months per year. No swIAV vaccinations against piglets were applied in selected commercial pig farms.

### 2.2. Trial Design and Sampling

Serological samples were collected from breeding animals (gilts and sows) and pigs aged 3 weeks to market age from different swIAV vaccinated and unvaccinated farms in regions with a high pig population density in Greece. In particular, a total of 1416 blood samples (gilts and sows of parity 1–6 and from pigs aged 4, 7, 12, 16, and 20 weeks) were collected from 40 farrowing farms in Greece between 2019 and 2023, grouped by their age (Table 1). All blood samples were collected by jugular puncture using disposable syringes and needles. Serum was collected from all blood samples after centrifugation (10 min at 3000× *g*) and stored at −80 °C for further laboratory analysis. The sampling strategy for this study was stratified random sampling within each farm. The strata in this case were nine predefined age groups of animals: 4 groups for gilts/sows (gilts, sows of parity 1–2, 3–4, 5–6) and 5 groups for pigs aged 4, 7, 12, 16 and 20 weeks of age). This setting allowed for comparisons of seroprevalence between different age groups because all age groups were adequately represented in the study. In addition, stratification by age was important in this study because of possible age-related differences in disease characteristics and animal conditions. In practice, this meant that a random sample of animals from each age group was selected at each farm. Sampling was conducted independently, and an equal number of animals from each age group was sampled at each farm, resulting in a data set that contained a balanced representation of age groups.

### 2.3. Serology

Sera were first analyzed with a commercially available ELISA for the detection of antibodies to influenza A nucleocapsids (ELISA, CIVTEST-Suis, Laboratorios Hipra SA, Amer, Girona, Spain). To evaluate the presence of anti-SIV antibodies, the commercial indirect ELISA kit CIVTEST suis Influenza^®^, Hipra (Amer, Girona, Spain) was used, as recommended by Simon-Grifé et al. [2]. The kit used antigens from the H1N1 strain of SIV, which according to the manufacturer also cross-reacts with other strains such as H1N2 and H3N2, although H1N1-positive sera were more frequently detected as positive than H1N2- or H3N2-positive ones [2]. Each sample was tested in duplicates, according to the manufacturer’s instructions, while positive and negative controls were provided in the kit. Optical density values (OD) were used to calculate IRPC (Relative Index × 100), which is obtained using the equation provided by the manufacturer:

Positive samples were those with an IRPC value >20.0, and consequently an IRPC value ≤20.0 was indicative of negative results. Both test performance and result interpretation were evaluated using the guidelines established by the kit manufacturer.

### 2.4. Data Analysis

This analysis used a logistic regression model with random intercepts, that is, a multilevel modeling approach that takes into account the hierarchical nature of the data. The outcome of interest was swIAV seroprevalence, a binary variable indicating whether an animal is seropositive or seronegative, based on the IRPC cutoff of 20.0. The primary predictor was age group, and both gilts/sows and pigs (see definition above) categories were included so that differences in seroprevalence in all age groups could be examined. Information on swIAV vaccination (present or absent) on the farm was also included as a predictor in the model. The random intercept for the region in which the farm is located was included in the model to adjust for region-specific effects that might influence seroprevalence.

We performed logistic regression analysis to estimate age-adjusted seroprevalence and the respective confidence intervals. Gilts from unvaccinated farms were selected as the reference group. The model provided odds ratios, which were then converted to probabilities to provide an intuitive interpretation of seroprevalence in each group compared with the reference group. The robustness of the model was checked using diagnostic methods for multilevel models, including testing for overdispersion, and examining residual plots. The age-adjusted overall seroprevalence and 95% confidence interval (CI) were estimated to account for the presence or absence of swIAV vaccination on the farm and potential confounding effects at the regional level. The analysis was performed in the R programming language [31] using the R packages lme4 [32] and boot [33] for analysis and the package ggplot2 [34] for generating the figures.

## 3. Results

Table 1 shows the number of animals sampled and the number of positive samples in each age group. Of the total 1416 animals sampled, 498 were seropositive, with the highest and lowest seroprevalence in sows of parity 5–6 and 12-week-old pigs, respectively. Twenty-seven (27) of the forty farms had a vaccination program against swIAV. Table 2 provides an overview of the number of sampled and positive animals per age group depending on whether swIAV vaccination was performed, while Table 3a,b show the number of sampled and positive animals per age group and the location for vaccinated and non-vaccinated farms, respectively. On swIAV-unvaccinated farms, a high seroprevalence was noticed in gilts/sows and finishing pigs. However, in swIAV-vaccinated farms, except for gilts/sows and finishing pigs, a high seroprevalence was noticed in weaned piglets (4 weeks of age) possibly due to maternally derived antibodies (MDA) from their vaccinated mothers (Table 2). In addition, the total seroprevalence in swIAV-vaccinated farms was higher in regions of North Greece and Crete (Table 3a), while in swIAV-unvaccinated farms, it was higher in regions of North and West Greece, as well as in Thessaly (Table 3b).

Table 4 summarizes the results of the implemented logistic regression model. It was found that all age groups, except sows of parity 1–2, were associated with swIAV seropositivity (*p*-value < 0.05). All age groups of pigs and the reference group were found to be negatively associated with swIAV seropositivity, i.e., these age groups were more likely to be seronegative, while sows of parity 3–4 and 5–6 were associated with swIAV seropositivity. In addition, the presence of swIAV vaccination on the farm was associated with swIAV seropositivity.

The overall prevalence of swIAV and its 95% confidence interval, adjusted for age group, swIAV vaccination implementation (presence or absence), and potential confounders at the regional level, were 35% (CI 95%, 33% to 36%).

## 4. Discussion

A previous study collecting data in 16 European countries between 2010 and 2013 reported that swIAV was detected in 31% of sampled commercial pig farms [5]. The predominant subtypes were the three European enzootic swIAV, H1N1 (53.6%), H1N2 (13%), and H3N2 (9.1%), and pandemic A/H1N1 2009 (H1N1pdm) (10.3%). A total of 13.9% of viruses were reassortants between these four lineages [5]. In Greece, our previous study showed that H1N1, H1N2, H3N2, and H1N1pdm viruses were detected in pig serum samples from apparently healthy pigs from 46 pig farms during 2010–2012 [30]. Antibodies against HIN1 (19.6%), H3N2 (28.3%), H1N1 + H3N2 (13%), H1N1pdm (6.5%), H1N1 + H1N1pdm (4.4%), and H3N2 + H1N1pdm (6.5%) were detected. The introduction of H1N1pdm in Greek pig farms may have been caused either by the importation of infected sows from countries with previous H1N1pdm outbreaks in pigs or by one or more anthropozoonotic events [30]. Our current study revealed a seroprevalence of 35% in serum samples, approximately 23% in swIAV-unvaccinated farms, and 54% in swIAV-vaccinated farms. The results of the current study indicate that swIAV is still circulating in commercial farrow-to-finish pig farms in Greece, both in vaccinated and unvaccinated farms.

The vaccination or infection status of sows can result in high swIAV strain-specific MDA levels, which can help to reduce swIAV circulation in both suckling and growing pigs [35,36]. MDA can persist in pigs for up to ten weeks and potentially lead to false positives [37]. Our results from swIAV-vaccinated farms confirm that MDA can last up to 12 weeks, with a decline of more than 50% between 4 and 7 weeks and even more up to 12 weeks. In swIAV-unvaccinated farms, MDA levels were lower in pigs at 4 and 7 weeks of age than in swIAV-vaccinated farms, and the decline at 4 to 7 weeks of age was smaller. However, MDA and swIAV could be expressed early after weaning in both farrowing and nursery farms [25,26,38,39,40]. Several studies reported that MDA (a) does not provide complete protection against swIAV infection and clinical symptoms [25,26,38,41,42,43,44], and (b) can impair an active humoral response to the presence of MDA at the time of primary infection, leading susceptible piglets to be reinfected, even with the same strain [21,26,36,37,40,43,45]. In our study, the highest seroprevalence in unvaccinated farms was found in breeding stock and finishing pigs (20 weeks old). Older sows (>3 parity) had higher seroprevalence than lactating sows and sows of parity 1–2, suggesting that swIAV circulates in unvaccinated farms and animals may be infected again with swIAV. An association with SIV seropositivity was found in all age groups except sows of parity 1–2 (*p*-value < 0.05). In particular, most of the age groups of pigs and the reference group (gilts in swIAV vaccinated farms) were more likely to test negative for swIAV antibodies (i.e., negatively associated with swIAV seropositivity), while sows of parity 3–4 and 5–6 were associated with swIAV seropositivity. In addition, the presence of swIAV vaccination on the farm was associated with swIAV seropositivity. The circulation of swIAV was also detected on vaccinated farms, as pigs older than 16 weeks of age had a higher seroprevalence than younger animals. Notably, the infection of animals older than 16 weeks is possible in vaccinated farms, since MDA decreases in finishing pigs at 3 to 4 months of age [46,47]. The higher observed seroprevalence in swIAV-unvaccinated farms (32%) in the region of Thessaly compared to swIAV-vaccinated farms (26%), based on our experience, might be due to the low level of swIAV vaccination status of farms in Thessaly. However, future studies, including sampling from an increased number of farms in Thessaly, are required. Nevertheless, our results underscore the importance of vaccination and field serological testing as an effective tool to prevent swIAV infections in commercial farrow-to-finish farms [48].

According to the swIAV seroprevalence in unvaccinated pig farms per region in Greece, the highest values were found in Northern and Western Greece and Thessaly. This result is particularly interesting for Northern and Western Greece because (a) the Western coast of Greece is an important migration route for African–Eurasian wild bird migration, being part of the Black Sea/Mediterranean migratory route [49,50,51,52,53], and (b) Western Greece is characterized by large-scale poultry farms and areas with high poultry density [54]. Avian influenza viruses (AIVs) are prevalent in wild birds worldwide and have been isolated from a variety of avian species [1,55], causing major problems in the industry [56]. Wild waterfowl are considered a natural reservoir for AIV [57]. In addition, there are highly pathogenic avian influenza (HPAI) strains that can infect both pigs and humans, which is an important global zoonotic and pandemic risk factor. Migratory birds are a natural reservoir for AIV; they carry different viral strains and exchange them along their migratory routes, resulting in antigenic drift and antigenic shift, which in turn results in the emergence of new HPAI viruses [58]. Poultry farms are known to play a critical role in the spread of AIV in an area, although wild birds are known to be the source of infection for domestic poultry and humans [59]. Biosecurity measures are important to limit the introduction of swIAV and should be strengthened on swine farms in areas with high densities of swine and poultry [60]. National and international regulatory agencies [61,62] and researchers worldwide [63] emphasize that improving biosecurity and surveillance practices and strategies is a priority in areas where AI vaccination is allowed under different circumstances [64].

In a recent meta-analysis, pigs were found to play a key role in the emergence of new types of epidemic zoonoses [65]. The swIAV seroprevalence rate in pigs and the swIAV infection trend in humans demonstrate the potential transmission of influenza from humans to pigs [66]. Swine workers, even with elevated pre-existing antibodies, are at high risk of infection with enzootic swIAV and more attention should be paid to the dynamics of influenza in pig herds and workers [67,68,69]. Investigating the factors that promote the persistence of swIAV in pig herds may aid in the development of strategies to eliminate swIAV prevalence and reduce the risk of zoonotic transmission to humans [70].

Widespread morbidity in swine herds negatively impacts animal welfare standards and economic performance, while pandemics of human influenza have occurred in pigs on several occasions. By effectively using the swIAV control measures available for swine, we can increase the economic productivity of swine farming while improving on-farm animal welfare standards, and avoiding the high social and financial costs of a pandemic. The application of control measures against swIAV in pig herds could reduce the risk of human pandemics and improve the health status and production of pig herds [71]. Vaccines are an important strategy to control swIAV in pigs, but their efficacy is not optimal, and they are underutilized. Recent studies highlight the value of swIAV vaccination in the pig industry, not only to limit virus replication in pigs but also to protect public health by limiting the emergence of new reassortants with zoonotic and/or pandemic potential threats [72]. The swine industry needs to increase vaccination rates in pigs to minimize the field circulation of the virus, reduce reassortments, and reduce the risk of pandemics in both humans and pigs [73]. The co-circulation of different swIAV strains in pigs may facilitate gene reassortment between strains, leading to the emergence of new circulating strains in pigs and strains with pandemic potential. Swine influenza epidemiology varies across and within countries due to factors such as climate, pig population, and farming practices [74]. However, future studies on swIAV are needed in Greece for a better understanding of swIAV epidemiology.

## 5. Conclusions

Our results indicate that swIAV continues to circulate in Greek pig farms, both in vaccinated and unvaccinated farms, with a seroprevalence of 35% (23% in unvaccinated and 54% in vaccinated farms). The highest seroprevalence in unvaccinated pig farms was found in the regions of northern and western Greece, as well as in Thessaly. The highest seroprevalence in unvaccinated farms was found in breeding and fattening pigs (at 20 weeks of age). Older sows (>3 parity) had higher seroprevalence than gilts and sows of parity 1–2, suggesting that swIAV also circulates in unvaccinated pig farms. Furthermore, MDA reduction in vaccinated farms was more than 50% between 4 and 7 weeks and even more at 12 weeks. The MDA content of pigs at 4 and 7 weeks of age was lower in unvaccinated farms than in vaccinated pig farms, and the reduction at 4 to 7 weeks of age was smaller. Our study underlines the importance of vaccination programs and serological testing to prevent swIAV infections in farrow-to-finish pig farms. However, future surveillance and genomic studies on swIAV in Greece are needed.

## Figures and Tables

**Table 1:** Data of the sampled farms containing a total of samples collected and samples tested positive per age group.

Age Group	No. of Animals Sampled	No. of Positive Samples	Seroprevalence within the Sample
Gilts	146	69	47%
Sows parity 1–2	161	85	53%
Sows parity 3–4	136	82	60%
Sows parity 5–6	134	83	62%
Pigs 4 weeks	181	60	33%
Pigs 7 weeks	171	27	16%
Pigs 12 weeks	180	18	10%
Pigs 16 weeks	155	31	20%
Pigs 20 weeks	152	43	28%
Total	1416	498	35%

**Table 2:** Data of the sampled farms containing a total of samples collected and samples tested positive per age group and absence (No) or application (Yes) of swIAV vaccination in the farm.

Age Group	swIAV Vaccination					
	No			Yes		
	No. of Samples	No. of Samples	Seroprevalence within the Sample	No. of Samples	No. of Samples	Seroprevalence within the Sample
Gilts	88	24	27%	58	45	78%
Sows, parity 1–2	97	33	34%	64	52	81%
Sows, parity 3–4	80	30	38%	56	52	93%
Sows, parity 5–6	81	36	44%	53	47	89%
Pigs 4 weeks	113	20	18%	68	40	59%
Pigs 7 weeks	103	11	11%	68	16	24%
Pigs 12 weeks	110	10	9%	70	8	11%
Pigs 16 weeks	88	8	9%	67	23	34%
Pigs 20 weeks	88	19	22%	64	24	38%
Total	848	191	23%	568	307	54%

**Table 3:** a. Data of the sampled vaccinated farms containing a total of samples collected and samples tested positive per age group and location (positive samples/number of samples collected (percentage)). b. Data of the sampled unvaccinated farms containing a total of samples collected and samples tested positive per age group and location (positive samples/number of samples collected (percentage)).

Age Group	Location					
	North Greece	Thessaly	West Greece	Central Greece	South Greece	Crete
a						
Gilts	10/13 (77%)	4/5 (80%)	14/16 (88%)	11/16 (69%)	1/3 (33%)	5/5 (100%)
Sows, parity 1–2	13/14 (93%)	0/5 (0%)	20/24 (83%)	13/13 (100%)	1/3 (33%)	5/5 (100%)
Sows, parity 3–4	13/13 (100%)	0/0 (0%)	21/23 (91%)	12/12 (100%)	1/3 (33%)	5/5 (100%)
Sows, parity 5–6	12/12 (100%)	0/0 (0%)	13/15 (87%)	16/18 (89&)	1/3 (33%)	5/5 (100%)
Pigs 4 weeks	12/13 (92%)	4/5 (80%)	8/24 (33%)	11/18 (61&)	0/3 (0%)	5/5 (100%)
Pigs 7 weeks	3/13 (23%)	0/5 (0%)	10/24 (42%)	0/18 (0%)	0/3 (0%)	3/5 (60%)
Pigs 12 weeks	1/13 (8%)	0/5 (0%)	4/27 (15%)	2/17 (12%)	0/3 (0%)	1/5 (20%)
Pigs 16 weeks	9/13 (69%)	1/5 (20%)	4/23 (17%)	6/18 (33%)	0/3 (0%)	3/5 (60%)
Pigs 20 weeks	12/13 (92%)	0/5 (0%)	2/22 (9%)	7/16 (44%)	0/3 (0%)	3/5 (60%)
Total	85/117 (73%)	9/35 (26%)	96/198 (48%)	78/146 (53%)	4/27 (15%)	35/45 (78%)
b						
Gilts	9/21 (43%)	2/11 (18%)	7/24 (29%)	3/13 (23%)	3/18 (17%)	0/1 (0%)
Sows, parity 1–2	14/22 (64%)	3/13 (23%)	9/30 (30%)	3/16 (19%)	2/11 (18%)	2/5 (40%)
Sows, parity 3–4	4/13 (31%)	7/10 (70%)	11/25 (44%)	2/15 (13%)	5/11 (45%)	1/6 (17%)
Sows, parity 5–6	6/18 (33%)	6/7 (86%)	15/28 (54%)	1/13 (8%)	4/11 (36%)	4/4 (100%)
Pigs 4 weeks	9/37 (24%)	2/16 (12%)	7/27 (26%)	0/14 (0%)	2/14 (14%)	0/5 (0%)
Pigs 7 weeks	3/37 (8%)	2/10 (20%)	4/22 (18%)	2/15 (13%)	0/14 (0%)	0/5 (0%)
Pigs 12 weeks	2/37 (5%)	3/16 (19%)	2/23 (9%)	3/15 (20%)	0/14 (0%)	0/5 (0%)
Pigs 16 weeks	1/27 (4%)	4/11 (36%)	1/19 (5%)	2/15 (13%)	0/11 (0%)	0/5 (0%)
Pigs 20 weeks	4/24 (17%)	6/16 (38%)	2/17 (12%)	5/15 (33%)	2/11 (18%)	0/5 (0%)
Total	52/236 (22%)	35/110 (32%)	58/215 (27%)	21/131 (16%)	18/115 (16%)	7/41 (17%)

**Table 4:** Estimate and 95% confidence intervals (CIs) for the effect of age group and swIAV vaccination from the random intercept logistic regression model.

Variable	Level	Coefficient (95% CI)	*p*-Value
Reference level	Gilts in swIAV-unvaccinated farms	0	-
Age group	Sows, parity 1–2	0.227 (−0.27; 0.72)	0.37
	Sows, parity 3–4	0.604 (0.08; 1.13)	0.02 *
	Sows, parity 5–6	0.712 (0.19; 1.24)	0.008 *
	Pigs 4 weeks	−0.738 (−1.23; −0.24)	0.003 *
	Pigs 7 weeks	−1.91 (−2.5; −1.34)	<0.001 *
	Pigs 12 weeks	−2.46 (−3.11; −1.85)	<0.001 *
	Pigs 16 weeks	−1.64 (−2.21; −1.08)	<0.001 *
	Pigs 20 weeks	−1.1 (−1.62; −0.56)	<0.001 *
swIAV Vaccination	Yes	1.76 (1.48; 2.04)	<0.001 *
	No	0	

* Statistical significance.

## Data Availability

All data generated for this study are presented within the manuscript.
